# Clinical features of brain metastases from hepatocellular carcinoma using gamma knife surgery

**DOI:** 10.1007/s00701-018-3504-1

**Published:** 2018-03-02

**Authors:** Akiyoshi Ogino, Tatsuo Hirai, Toru Serizawa, Atsuo Yoshino

**Affiliations:** 1 Department of Neurological Surgery, Nihon University School of Medicine, 30-1 Oyaguchi-Kamimachi, Itabashi-ku, Tokyo, 173-8610 Japan; 2 Tokyo Gamma Unit Center, Tsukiji Neurological Clinic, Tokyo, Japan

**Keywords:** Hepatocellular carcinoma, Brain metastases, Gamma knife surgery, Hepatitis B virus

## Abstract

**Background:**

Brain metastases from hepatocellular carcinoma (HCC) are rare, but their incidence is increasing because of developments in recent therapeutic advances. The purpose of this study was to investigate the characteristics of brain metastases from HCC, to evaluate the predictive factors, and to assess the efficacy of gamma knife surgery (GKS).

**Method:**

A retrospective study was performed on patients with brain metastases from HCC who were treated at Tokyo Gamma Unit Center from 2005 to 2014.

**Results:**

Nineteen patients were identified. The median age at diagnosis of brain metastases was 67.0 years. Fifteen patients were male and four patients were female. Six patients were infected with hepatitis B virus (HBV). Two patients were infected with hepatitis C virus (HCV). Eleven patients were not infected with HBV or HCV. The median interval from the diagnosis of HCC to brain metastases was 32.0 months. The median number of brain metastases was two. The median Karnofsky performance score at first GKS was 70. The median survival time following brain metastases was 21.0 weeks. Six-month and 1-year survival rates were 41.2 and 0%, respectively. One month after GKS, no tumor showed progressive disease. The HBV infection (positive vs. negative) was significantly associated with survival according to univariate analysis (*p* = 0.002).

**Conclusions:**

The patients having brain metastases from HCC had poor prognosis and low performance state. Therefore, GKS is an acceptable option for controlling brain metastases from HCC because GKS is noninvasive remedy and local control is reasonable.

## Introduction

Hepatocellular carcinoma (HCC) is one of the most frequent malignant tumors in the world [20]. The incidence of HCC is the highest in East and South-East Asia and Sub-Saharan Africa, as a result of the high prevalence of hepatitis B virus (HBV) and hepatitis C virus (HCV) [15]. In Japan, HCC is the sixth common cancer and the fifth cause of cancer deaths. Brain metastases from HCC are very rare, with their incidence ranging from 0.2 to 2.2% [3, 10, 12, 17]. However, recent studies have reported an increased incidence of brain metastases from HCC [18]. The reason behind this is recent therapeutic advances in surgical techniques and new methods, such as transarterial chemoembolization (TACE) and percutaneous ethanol injection (PEI), which have prolonged the life time of the patients. Therefore, there are more opportunity chances of metastases to the brain [16]. Because of the advanced stage at diagnosis of brain metastases from HCC, the prognosis is quite poor [3, 6, 8]. Survival rate at 1 year is usually less than 10% [3, 6]. Because gamma knife surgery (GKS) for brain metastases is minimally invasive and has good control rate, it is an effective remedy [1, 2]. Nevertheless, to our knowledge, there are only few reports, and all are from East Asia. Many patients with HCC in East Asia were infected with HBV [3, 5, 9, 13]. However, in Japan, the main cause of HCC is HCV infection [7] and there are no reports regarding this. In this study, we investigated the clinical features of patients with brain metastases from HCC in Japan, to compare with previous studies, to identify the prognostic factors affecting patient survival, and to evaluate the efficacy of GKS for local tumor control of brain metastases.

## Materials and methods

### Patients

A total of 4178 patients with brain metastases were treated with GKS from November 2005 to February 2014 at the Tokyo Gamma Unit Center. The database of the hospital was searched to identify patients with brain metastases from HCC, and 19 patients treated with GKS were identified. Diagnoses in all patients were made using gadolinium-enhanced magnetic resonance imaging (MRI) with or without contrast-enhanced computed tomography.

Stereotactic radiation was performed using a Leksell Gamma Knife C (Elekta Instruments AB) until November 2012 and with a Leksell Gamma knife Perfexion (Elekta Instruments AB) from December 2012 onward. All patients underwent thin-slice gadolinium-enhanced MRI after placing the Leksell Model G stereotactic frame (Elekta Instruments AB), and the treatment plans were developed using GammaPlan (Elekta Instruments AB). Follow-up MRI was performed every 1–3 months after GKS. We measured tumor size and assessed the response to GKS using the Response Evaluation Criteria in Solid Tumors guideline [19]. Since the prognosis of brain metastasis from HCC was poor, we were able to measure the size of only 16 tumors in 1 month after GKS.

To evaluate the predictive factors for survival after brain metastases from HCC, the following characteristics were reviewed: age at the diagnosis of brain metastases, sex, HBV infection, HCV infection, Karnofsky performance score (KPS) at first GKS, recursive partitioning analysis (RPA) at first GKS, interval from the diagnosis of HCC to brain metastases, number of brain metastases at first GKS, and total volume of brain metastases at first GKS.

### Statistical analysis

The data was analyzed using a personal computer running SPSS Statistics, version 23.0 (IBM, New York, USA). Survival was calculated from initial diagnosis of brain metastases until death. The Kaplan–Meier method was used to calculate survival distributions. Differences in survival were analyzed using a log-rank test. The relationships between the various parameters were analyzed statistically using the Mann–Whitney *U* test or the *x*^2^ test as appropriate. The significance level chosen was *p* < 0.05.

## Results

### Patient characteristics

In total, 4178 patients with brain metastases were treated with GKS at the Tokyo Gamma Unit Center from November 2005 to February 2014. Nineteen (0.45%) of these patients had brain metastases from HCC. The median age at the diagnosis of HCC and brain metastases was 64.0 years (range, 38–78 years) and 67.0 years (range, 39–83 years), respectively. Fifteen patients were males and four patients were females. Six patients (31.6%) were infected with HBV. Two patients (10.5%) were infected with HCV. Eleven patients (57.9%) were not infected with HBV and HCV. The median KPS was 70 (range, 50–100) at first GKS, and RPA classification at first GKS was class II in ten patients and class III in nine patients. The median interval from the diagnosis of HCC to brain metastases was 32.0 months (range, 0–72 months). The median number of brain metastases was 2.0 (range, 1–19), and the median total volume at first GKS was 12.6 cm^3^ (range, 1.1–47.6 cm^3^). The clinical characteristics of this study are shown in Table 1.

**Table 1:** Characteristics of the 19 patients

Case no.	Age (years)	Sex	HBV	HCV	KPS at first GKS	RPA at first GKS	Symptoms	Interval to brain metastases (months)	Number of brain metastases at first GKS	Total volume of brain metastases at first GKS (cm^3^)	Other metastases	Survival time (weeks)
1	73	Male	–	–	50	III	EW	19	1	5.2	Lung	38
2	67	Male	+	–	50	III	EW	20	1	7.8	PALN	5
3	58	Female	+	–	100	II	None^a^	46	2	1.1	Lung	7
4	75	Male	–	–	50	III	EW	39	1	24.3	Lung	17
5	45	Male	–	–	90	II	EW	34	1	7.3	Lung	11
6	57	Male	–	–	90	II	None^a^	72	1	8.3	Lung	47
7	60	Male	–	–	50	III	DC	17	10	16.6	Lung	6
8	78	Female	–	+	50	III	EW	0	1	5.1	NA	28
9	44	Male	+	–	60	III	VD	30	3	47.6	Lung	8
10	66	Male	+	–	80	II	EW	52	9	22.3	Lung, bone	16
11	70	Male	–	–	80	II	SD	NA	19	13.1	Lung	6
12	72	Male	–	–	60	III	EW	47	1	14.8	Lung	46
13	39	Male	+	–	50	III	EW	17	1	16.6	Lung, CLN	2
14	51	Female	+	–	90	II	None^a^	11	5	2.5	Lung, liver, and bone	2
15	65	Male	–	–	50	III	NA	60	2	32.0	Lung	28
16	83	Female	–	+	70	II	VD	63	2	5.8	NA	5
17	71	Male	–	–	90	II	EW	0	1	8.1	NA	21
18	70	Male	–	–	90	II	Seizure	27	3	23.4	Lung	11
19	79	Male	–	–	70	II	Seizure	48	6	12.6	NA	32

*+*, positive; *−*, negative; *EW*, extremity weakness; *PALN*, para-aortic lymph node; *DC*, disturbance of consciousness; *NA*, not acquired; *VD*, visual field defect; *SD*, sensory disturbance; *CLN*, cervical lymph node

^a^Brain metastases detected after MRI and/or CT

### Treatment

One patient was treated with surgical resection and stereotactic radiotherapy prior to GKS (case 12), and one patient was treated with surgical resection before GKS (case 15). In addition, one patient was treated with surgical resection because of intra-tumor hemorrhage after GKS, but the tumor had not carried out GKS yet by a new lesion (case 16). Sixteen patients were treated with GKS alone for brain metastases. The treatment delivered to the tumor margin ranged from 12.5 to 22.3 Gy (median, 20.1 Gy). The maximum tumor dose ranged from 25.0 to 51.5 Gy (median, 40.2 Gy). The number of shots ranged from 2 to 40 (median, 14). The prescription isodose ranged from 35 to 60% (median, 50%). The coverage ranged from 0.98 to 1.0 (median, 1.0). The conformity index ranged from 0.54 to 0.86 (median, 0.72). The treatment characteristics of this study are shown in Table 2.

**Table 2:** Treatment characteristics of the 19 patients at first GKS

Case no.	Prescription dose (Gy)	Reference dose (Gy)	Number of shots	Prescription isodose (%)	Coverage	Conformity index
1	20.6	41.2	4	50	1.0	0.61
2	20.3	45.0	4	45	1.0	0.69
3	21.5	43.0	2	50	1.0	0.55
4	14.0	28.0	23	50	1.0	0.83
5	21.5	43.0	29	50	1.0	0.85
6	20.0	40.0	14	50	0.98	0.79
7	21.1	42.2	17	50	0.99	0.74
8	20.0	40.0	17	50	1.0	0.86
9	20,1	40.2	32	50	1.0	0.72
10	22.3	37.2	10	60	1.0	0.64
11	20.1	40.3	20	50	1.0	0.75
12	12.5	25.0	13	50	1.0	0.68
13	17.5	50.0	40	35	1.0	0.83
14	20.0	40.0	8	50	1.0	0.54
15	20.0	40.0	24	50	1.0	0.63
16	20.0	40.0	9	50	1.0	0.63
17	19.5	48.7	14	40	1.0	0.75
18	20.6	51.5	13	40	1.0	0.78
19	20.1	40.2	17	50	1.0	0.71

### Outcome and prognostic analysis

At the last follow-up, three patients were alive and 16 died. But these patients who were alive were not followed. The overall median survival from brain metastases was 21.0 weeks (range, 2–47 weeks). The 6-month and 1-year survival rates from the diagnosis of brain metastases were 41.2 and 0%, respectively. The main causes of death were extracranial diseases: deterioration of HCC, nine cases (case 1, 2, 5, 6, 7, 10, 16, 17, and 18); respiratory failure due to deterioration of lung metastases, two cases (case 9, and 13); and undetermined cause of death, five cases (case 3, 11, 12, 15, and 19). Sixteen tumors were able to be measured for tumor size at 1 month after GKS; seven tumors showed a partial response (PR) and nine showed a stable disease (SD). One SD tumor at 1 month after GKS became progressive disease at 3 months after the procedure.

Age, sex, HCV infection, KPS, RPA, interval to brain metastases, number of brain metastases, and total volume of brain metastases were all not associated with survival after brain metastases. However, HBV infection was associated with survival as suggested by a univariate analysis. The median overall survival for 13 patients who were not infected with HBV was significantly longer than that of the six patients infected with HBV (32.0 vs. 7.0 weeks; *p* = 0.002). The number of brain metastases was remarkably not significantly associated with survival, but the median overall survival for nine patients with only a single metastasis tended to be longer than that of the ten patients with multiple metastases (38.0 vs. 11.0 weeks; *p* = 0.069). The total volume of brain metastases was not associated with survival; however, the median overall survival for 12 patients with total volume of brain metastases < 15 cm^3^ tended to be longer than that of the seven patients with total volume of brain metastases ≧ 15 cm^3^ (32.0 vs. 11.0 weeks; *p* = 0.086) (Table 3).

**Table 3:** Univariate analysis of survival after diagnosis of brain metastases

	No. of patients	Median survival (weeks)	Log-rank dp value
Age at diagnosis of brain metastases			
< 60 years	6	8.0	0.778
≥ 60 years	13	28.0	
Sex			
Male	15	16.0	0.650
Female	4	35.0	
HBV			
Positive	6	7.0	0.002
Negative	13	32.0	
HCV			
Positive	2	35.0	0.338
Negative	17	16.0	
KPS at first GKS			
< 80	11	32.0	0.709
≥ 80	8	11.0	
RPA at first GKS			
II	10	16.0	0.924
III	9	28.0	
Interval to brain metastases			
< 2 years	7	21.0	0.401
≥ 2 years	11	28.0	
Number of brain metastases at first GKS			
Single	9	38.0	0.069
Multiple	10	11.0	
Total volume of brain metastases at first GKS			
< 15 cm^3^	12	32.0	0.086
≥ 15 cm^3^	7	11.0	

*RPA*, recursive partitioning analysis

## Discussion

Data from previous studies have indicated the following: the median age at diagnosis of brain metastases was from 48.5 to 56.1 years old, the percentage of males ranges from 75.8 to 87.7, the percentage of HBV-related HCC ranged from 73.2 to 91.6%, the percentage of HCV-related HCC ranged from 0 to 6.9%, the median interval from diagnosis of HCC to brain metastases ranged from 15.0 to 29.5 months, and the median overall survival from the diagnosis of brain metastases ranged from 4.3 to 20.0 weeks (Table 4) [3, 6, 8, 9, 13, 14, 21]. The results from our patients support the data of the median interval from diagnosis of HCC to brain metastases and the percentage of males. In the present study, the median age at diagnosis of brain metastases was 67.0 years, and the percentage of HBV related HCC was remarkably low at 31.6%. These substantial differences from the norm were likely due to the specific cause of HCC in our patients. In Japan, the main cause of HCC is HCV infection, and approximately 67.7% patients with HCC were also infected with HCV [7]. The percentage of HCC patients who also had HBV infection was approximately 15% [7]. Furthermore, several studies in the literature regarding brain metastases from HCC have been based on studies in East Asia. In East Asia, the percentage of HCC patients that had HBV infections has been reported to be approximately 80 to 90% [3, 5, 9, 13]. HCC patients with HBV are generally younger than those not infected with HBV [11]. Thus, in the present study, HCC associated with HBV may be relatively low, and the median age of our patients may be older. In Japan, two-third patients with HCC were infected with HCV; however, only 10.5% patients with brain metastases from HCC were infected with HCV in this study. There were many patients with HCC who were not infected with both HBV and HCV in this study. In patients with HCC infected with HCV, brain metastases may be rare; however, in patients with HCC not infected with HBV and HCV, brain metastases may be not rare. The median overall survival from the diagnosis of brain metastases in this study was a little longer than that in the previous study. The reason was the lesser number of patients with HCC infected with HBV having poor prognosis [4].

**Table 4:** Literature review of brain metastases from hepatocellular carcinoma

Authors	Study period	Number of patients	Percentage incidence	Median age at the brain metastasis	Percentage of man	HBV infection (%)	HCV infection (%)	Median interval to brain metastasis (months)	Percentage of intracranial hemorrhage relevance to tumor	Median survival from brain metastasis (weeks)
Choi HJ et al.	1995–2006	62	0.9^a^	54	75.8	85.5	3.2	18.2	54.8	6.8
Han JH et al.	1998–2011	32	2.6^b^	54^c^	87.5	84.4	–	26.0	76.0	11.3^d^
Jiang XB et al.	1994–2009	41	0.5^a^	48.5	80.5	73.2	0	15.0	46.3	12.0
Kim KS et al.	2000–2011	95	–	56.1	86.3	91.6	5.3	29.5	74.7	12.0
Park ES et al.	1993–2012	73	–	52.5^c^	87.7	84.9	6.9		47.9	16.0
Park TY et al.	2004–2012	59	–	52.2^c^	83.1	–	–	–	33.9	4.3
Xu Q et al.	–	14	–	53^c^	85.7	85.7	0	–	–	20.0
Present study	2005–2014	19	0.45^b^	67.0	78.9	31.6	10.5	32.0	–	21.0

^a^Percentage incidence of hepatocellular carcinoma

^b^Percentage incidence of brain metastases

^c^Mean age

^d^Median survival after Stereotactic radiosurgery

Brain metastases from HCC manifests at a late stage, and most patients also have coagulopathy due to liver cirrhosis. Survival is generally poor, regardless of treatment including surgical resection of brain metastases, chemotherapy, radiotherapy, and various combinations of these treatments [6]. Kim et al. reported that multi-modality treatment resulted in longer survival than single-modality treatment (WBRT only, radiosurgery only, or surgical resection only) or conservative management. The median survival after multi-modality treatment and single-modality treatment were 10.6 and 2.6 months, respectively [9]. Choi et al. reported that combination therapy employing both surgical resection and WBRT resulted in a relatively good prognosis [3]. However, these studies were based retrospectively, some patients were receiving multi-modality treatments, and there may have been substantial selection bias. In our study, statistical analysis for the efficacy of combination treatment was not performed because only two patients underwent combination therapy (cases 12 and 16). Furthermore, the efficacy of systemic chemotherapy for brain metastases from HCC remains unknown. Despite considerable effort, we could not find authoritative studies on this subject.

There are few reports including a considerable number of patients that investigate the effect of GKS in dealing with brain metastases from HCC, and our study is the third largest in this respect [5, 13]. Park et al. reported on 73 patients with brain metastases from HCC who were treated with GKS [13]. The median survival after GKS was 16.0 weeks, and the 24-week survival rate was 26.0%. The estimated rate of local tumor control was 79.6% at 3 months after GKS. These results suggested that GKS was a noninvasive approach that may provide a valuable option for treating patients with brain metastases from HCC.

Several studies have identified significant predictive factors for brain metastases from HCC [3, 8, 9, 13, 21]. These included the number of brain metastases, RPA, Child–Pugh classification, and tumor bleeding. In our study, HBV-related HCC was a factor significantly associated with shorter survival (*p* = 0.002). The following parameters were compared between patients infected with HBV and those not infected with HBV: age, sex, KPS at first GKS, RPA at first GKS, interval from diagnosis to brain metastases, the number of brain metastases at first GKS, and the total volume of brain metastases at first GKS. Of these, only age showed significant difference. Unfortunately, Child–Pugh classification, tumor bleeding data, state of primary lesion, and data of liver function could not be obtained from medical records. Therefore, we cannot imply that patients infected with HBV have poor prognosis. Our study is apparently the first to report that patients with HBV-related HCC have a relatively poor prognosis. Up to this point, only a limited number of patients with brain metastases from HCC have been treated with GKS, and prospective data are lacking. However, the thrust of the existing literature in this area indicates that the local control rate of GKS for brain metastases from HCC is reasonable, and has few side effects. Many patients with brain metastases from HCC have poor general health states and low KPS. GKS may be an acceptable option for the control of brain lesions in HCC patients.

## Conclusions

Brain metastases from HCC are rare, but their incidence is becoming more frequent as patient survival has improved by recent therapeutic advances in surgical techniques and other improved treatments, such as TACE and PEI. In our study, the median survival after brain metastases was 16.0 weeks. There were no PD tumors at 1 month after GKS. HBV infection was an important factor predicting survival. Overall, our results suggest that GKS is an acceptable option for the control of brain metastases from HCC.
